# Intronic Haplotypes in the *GBA* Gene Do Not Predict Age at Diagnosis of Parkinson's Disease

**DOI:** 10.1002/mds.28616

**Published:** 2021-05-19

**Authors:** Marco Toffoli, Abigail Higgins, Chiao Lee, Sofia Koletsi, Xiao Chen, Michael Eberle, Fritz J. Sedlazeck, Stephen Mullin, Christos Proukakis, Anthony H.V. Schapira

**Affiliations:** ^1^ Department of Clinical and Movement Neurosciences University College London Queen Square Institute of Neurology London United Kingdom; ^2^ Research and Technology Development Illumina Inc. San Diego California USA; ^3^ Human Genome Sequencing Center Baylor College of Medicine Houston Texas USA; ^4^ Institute of Translational and Stratified Medicine University of Plymouth Peninsula School of Medicine Plymouth United Kingdom

**Keywords:** Parkinson's, *GBA*, haplotypes, intronic variants, genetics

## Abstract

**Background:**

*GBA* mutations are a common risk factor for Parkinson's disease (PD). A recent study has suggested that *GBA* haplotypes, identified by intronic variants, can affect age at diagnosis of PD.

**Objectives:**

In this study, we assess this hypothesis using long reads across a large cohort and the publicly available Accelerating Medicines Partnership–Parkinson's Disease (AMP‐PD) cohort.

**Methods:**

We recruited a PD cohort through the Remote Assessment of Parkinsonism Supporting Ongoing Development of Interventions in Gaucher Disease study (RAPSODI) and sequenced *GBA* using Oxford Nanopore technology. Genetic and clinical data on the full AMP‐PD cohort were obtained from the online portal of the consortium.

**Results:**

A total of 1417 participants were analyzed. There was no significant difference in age at PD diagnosis between the two main haplotypes of the *GBA* gene.

**Conclusions:**

*GBA* haplotypes do not affect age at diagnosis of PD in the two independent cohorts studied. © 2021 The Authors. *Movement Disorders* published by Wiley Periodicals LLC on behalf of International Parkinson and Movement Disorder Society

Mutations in the *GBA* gene are an important risk factor for the development of Parkinson's disease (PD).^1^ The prevalence of *GBA* mutations in PD varies according to the population studied and whether the analysis includes all coding variants or only the most common; in general it ranges between 5% and 10% of sporadic PD cases.^2^ The Ashkenazi Jewish population is an exception, with a prevalence of *GBA* mutations as high as 30% in sporadic PD cases.^3^ The lifetime risk of development of PD in exonic *GBA* mutation carriers is estimated at 5% to 30%,^4^ and it is not clear what factors contribute to this incomplete penetrance. Moreover, reduced activity of the enzyme encoded by *GBA*, glucocerebrosidase, is observed in PD brains without coding *GBA* mutations.^5^

A recent study explored the hypothesis that intronic variants in the *GBA* gene might contribute to the risk of PD.^6^ Deep intronic variants are not commonly regarded as pathogenic as they do not result in amino acid changes in proteins. Nonetheless, some deep intronic variants have been linked directly to genetic disorders, such as Gaucher disease.^7^, ^8^ Two common haplotypes were identified, differentiated by three intronic single nucleotide polymorphisms in *GBA*. These correspond to the previously reported 1.1+ and 1.1− haplotypes.^9^, ^10^ These haplotypes had a significant effect on age at onset and age at diagnosis of PD.

In this article, we analyzed our cohort of patients with PD and the publicly available Accelerating Medicines Partnership–Parkinson's Disease (AMP‐PD) cohort to try to replicate these findings.

## Patients and Methods

### Recruitment of Participants

Patients with PD were recruited through the Remote Assessment of Parkinsonism Supporting Ongoing Development of Interventions in Gaucher Disease study (RAPSODI) (http://rapsodistudy.com) an online cohort study that recruits and genotypes people with PD through a dedicated portal (http://pdfrontline.com). After signing an online consent form, participants were assessed remotely, and demographic and clinical information was recorded. Participants provided saliva DNA for analysis. The London Queen Square Research Ethics Committee approved the project.

### DNA Extraction and Sequencing

Saliva was collected using the Oragene DNA OG‐500 kit (DNA Genotek), and DNA was extracted according to the manufacturer's protocol. Sequencing of long *GBA* amplicons was carried out using Oxford Nanopore Technologies as described previously.^11^ Sequencing data were generated using MinKNOW, basecalled with Guppy (both available via the Nanopore community site; https://community.nanoporetech.com), and aligned to hg38 with NGMLR.^12^ Variants were called using Clair^13^ and phased with Whatshap.^14^ Haplotypes were identified using the R package Haplotypes (R Foundation for Statistical Computing). A detailed pipeline is reported in Figure S1.

### AMP‐PD Cohort Data

Clinical and sequencing data were downloaded from the AMP‐PD initiative website (https://amp-pd.org). Full details on data collection can be found on the website. Age at diagnosis, sex, and the *GBA* gene mutation calls for cases diagnosed as “idiopathic PD” or “Parkinson's disease” in the AMP‐PD cohort were downloaded on July 20, 2020 (version 2019_v1release_1015).

### Haplotype Definition

The full *GBA* gene sequence was analyzed for all participants with PD from both the RAPSODI and AMP‐PD cohorts. Carriers of coding *GBA* variants were excluded from the analysis. Each *GBA* allele was assigned to one of two haplotypes.^6^ Haplotype A was identified by alternate genotype at three intronic variants (rs9628662, rs762488, and rs2009578), whereas haplotype B was identified by the reference genotype at these variants. The minor allele population frequencies in non‐Finnish Europeans in the Genome Aggregation Database (GnomAD) data (https://gnomad.broadinstitute.org) are 0.295, 0.294, and 0.287, respectively. Participants who carried at least one allele that did not fall into this classification or for which quality of the alignment at any of the three positions was not good enough for confident calling were excluded from the analysis.

### Statistical Analysis

R version 4.0.2 was used for all statistical analyses. To assess the effect of haplotypes on age at diagnosis of PD, two models were investigated: an additive model and a dominant model of haplotype B. For the additive model, linear regression was used, with the number of alleles carrying haplotype B as the dependent variable. For the dominant model of haplotype B, participants were divided into two groups according to whether they carried at least one allele with haplotype B, and ANOVA was used for the analysis.

## Results

### Participants and Genotypes

We analyzed 1417 patients, of whom 100 were recruited through RAPSODI, and the remainder were from AMP‐PD. More than 100 unique haplotypes were identified (Figure S2), and the overall allelic frequency of each haplotype was 0.302 for haplotype A and 0.691 for haplotype B. The number of participants carrying each haplotype, genotypes, and mean age at diagnosis of PD are reported in Table 1. Of note, five (0.5%) participants in the AMP‐PD cohort carried at least one allele that was not classifiable in either of the two haplotypes, and were excluded from the analysis. Moreover, five samples in the RAPSODI cohort (5.0%) were not classifiable into one of the two haplotypes. Upon visual inspection of the sequencing data, the quality was not adequate for unequivocal haplotype assignment, and they were thus also excluded. Ethnic backgrounds in the two cohorts are reported in Tables S1 and S2.

**TABLE 1:** *GBA* haplotypes and age at diagnosis of PD

	All ages								
	RAPSODI cohort			AMP‐PD cohort			Merged cohorts (RAPSODI + AMP‐PD)		
Haplotype	Number of participants	Mean age at diagnosis	Median age at diagnosis	Number of participants	Mean age at diagnosis	Median age at diagnosis	Number of participants	Mean age at diagnosis	Median age at diagnosis
Homozygous haplotype A	10	61.5	59.5	131	60.1	61.0	141	60.2	61.0
Heterozygous	33	59.9	60.0	540	59.5	61.0	573	59.5	61.0
Homozygous haplotype B	52	61.3	61.0	641	60.1	61.0	693	60.2	61.0
Other	5			5			10		
Total	100			1317			1417		
*P* value additive model	0.7292			0.5819			0.528		
*P* value dominant haplotype B	0.7892383			0.7981861			0.7552578		

	Age at diagnosis > 50 years								
	RAPSODI cohort			AMP‐PD cohort			Merged cohorts (RAPSODI + AMP‐PD)		
Haplotype	Number of participants	Mean age at diagnosis	Median age at diagnosis	Number of participants	Mean age at diagnosis	Median age at diagnosis	Number of participants	Mean age at diagnosis	Median age at diagnosis
Homozygous haplotype A	10	61.5	59.5	104	64.0	64.0	114	63.8	64.0
Heterozygous	29	61.8	61.0	445	63.0	63.0	474	62.9	63.0
Homozygous haplotype B	48	62.7	61.0	526	63.6	63.0	574	63.5	63.0
Other	4			5			9		
Total	91			1080			1171		
*P* value additive model	0.5504			0.7416			0.6431		
*P* value dominant haplotype B	0.7378356			0.3714943			0.4654556		

PD, Parkinson's disease; AMP‐PD, Accelerating Medicines Partnership–Parkinson's Disease.

### Haplotypes and Age at Diagnosis of PD

**FIG. 1.**
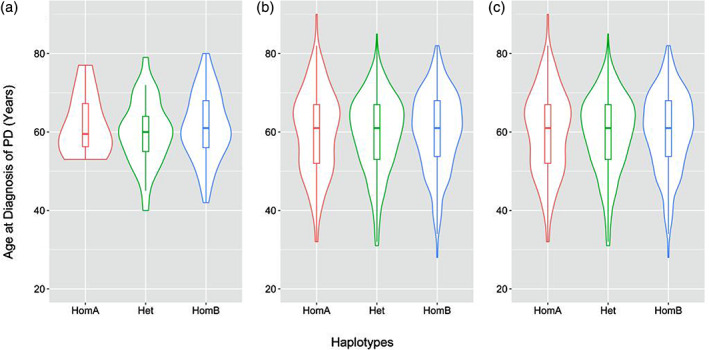
Distribution of age at diagnosis of PD symptoms by *GBA* haplotypes. The boxes show the medians, and the hinges are the first and third quartiles (25th and 75th percentiles): (**a**) RAPSODI cohort, (**b**) AMP‐PD cohort, and (**c**) RAPSODI and AMP‐PD cohorts together. RAPSODI, Remote Assessment of Parkinsonism Supporting Ongoing Development of Interventions in Gaucher Disease study;AMP‐PD, Accelerating Medicines Partnership–Parkinson's Disease; Het, heterozygous; HomA, homozygous haplotype A; HomB, homozygous haplotype B; PD, Parkinson's disease.

Mean and median age at diagnosis of PD are reported in Table 1 and shown in Figure 1. After considering both an additive model and a dominant effect of haplotype B model, age at diagnosis of PD was not significantly different in the RAPSODI and AMP‐PD cohorts separately. There was also no significant difference after merging the two cohorts together (*P* value > 0.3 for both the additive model and dominant haplotype B model).

Because a significant number of participants with early‐onset PD (EOPD) can carry mutations in other PD‐causing genes,^15^ we repeated the analysis after removing all participants with an age at diagnosis younger than 50. Following this adjustment, there were 91 participants in the RAPSODI cohort and 883 participants in the AMP‐PD cohort. Still, no significant effect of the two *GBA* haplotypes on age at diagnosis of PD was observed. Data on this additional analysis are reported in Table S1.

## Discussion

In this article, we attempted to validate the recent report that common haplotypes, identified by deep intronic variants in the *GBA* gene, could affect age at diagnosis of PD.^6^ This hypothesis is intriguing as it could help explain the reduced penetrance of *GBA* mutations and the role of intronic variants in the pathogenesis of PD.

To this end, we analyzed our original cohort, generated through the RAPSODI portal, and the publicly available AMP‐PD cohort. We investigated both an additive effect of the haplotypes and a dominant effect of haplotype B, but did not observe any effect of haplotypes on age at diagnosis of PD.

Both the RAPSODI and AMP‐PD cohorts included some participants who received a diagnosis of PD earlier than age 50 and would thus be classified as EOPD. Because a significant number of patients with EOPD can carry variants in other PD‐causing genes, we repeated the analysis after excluding all patients with EOPD. We still did not see any significant differences in age of onset between the different haplotypes.

The RAPSODI and AMP‐PD cohorts are similar in their ethnic profiles. In RAPSODI, 96% of participants identified themselves as “White UK,” and in the AMP‐PD cohort 93% of participants identified as “White.” The remarkably similar minor allele frequencies in our cohort to the European GNOMAD samples support this ethnic classification. It is possible that the cohort studied by Schierding et al.^14^ has a different balance of ethnic backgrounds, which might explain in part the discrepancy of results, although this information was not provided. Moreover, the inclusion of EOPD in the article by Schierding et al. might have influenced the results.

One limitation of our study is that we could not assess age at onset of PD symptoms, which had also been reported as variable by haplotype, as this was not captured in the RAPSODI and AMP‐PD cohorts.

Our study does not exclude a possible role for intronic variants. Although it is true that the majority of alleles could be grouped into one of the two main haplotypes according to their genotypes in three deep intronic variants, more than 50 unique intronic haplotypes were identified (Figure S2), and the role of each single intronic variant in PD might extend beyond that of these haplotypes and merits further study.

## Conclusions

In this study, we were not able to confirm a role for common *GBA* haplotypes in determining age at diagnosis of PD.

## Author Roles

(1) Research Project: A. Conception, B. Organization, C. Execution; (2) Statistical Analysis: A. Design, B. Execution, C. Review and Critique; (3) Manuscript: A. Writing of the First Draft, B. Review and Critique.

M.T.: 1A, 2B, 2C, 3A

C.P.: 1A, 3B

X.C.: 1C, 2C, 3B

A.H.: 1C, 3B

C.L.: 1C, 3B

S.M.: 3B

S.K.: 1C, 3B

M.E.: 3B

F.J.S.: 3B

A.H.V.S.: 1A, 3B

## Financial Disclosures

M.T. is a recipient of a student bourse from University College London. C.P., A.H., C.L., and S.K. are employees of University College London. S.M. works for the NHS. X.C. and M.E. are employees of Illumina Inc. F.J.S. is an employee of Baylor College of Medicine. A.H.V.S. is an employee of University College London and a consultant to Kyowa.

## Supporting information

**Appendix S1**: Supporting information
