# Supplementary material for: Intronic Haplotypes in the GBA Gene Do Not Predict Age at Diagnosis of Parkinson's Disease
Source: Mov Disord. 2021 May 19;36(6):1456–60. doi: 10.1002/mds.28616 (PMC8436748; doi:10.1002/mds.28616)
Supplement: Supplementary file 1 — Appendix S1: Supporting information [file MDS-36-1456-s001.docx]

**Supplementary figure 1:** Pipeline for sequencing and haplotype analysis of RAPSODI samples.


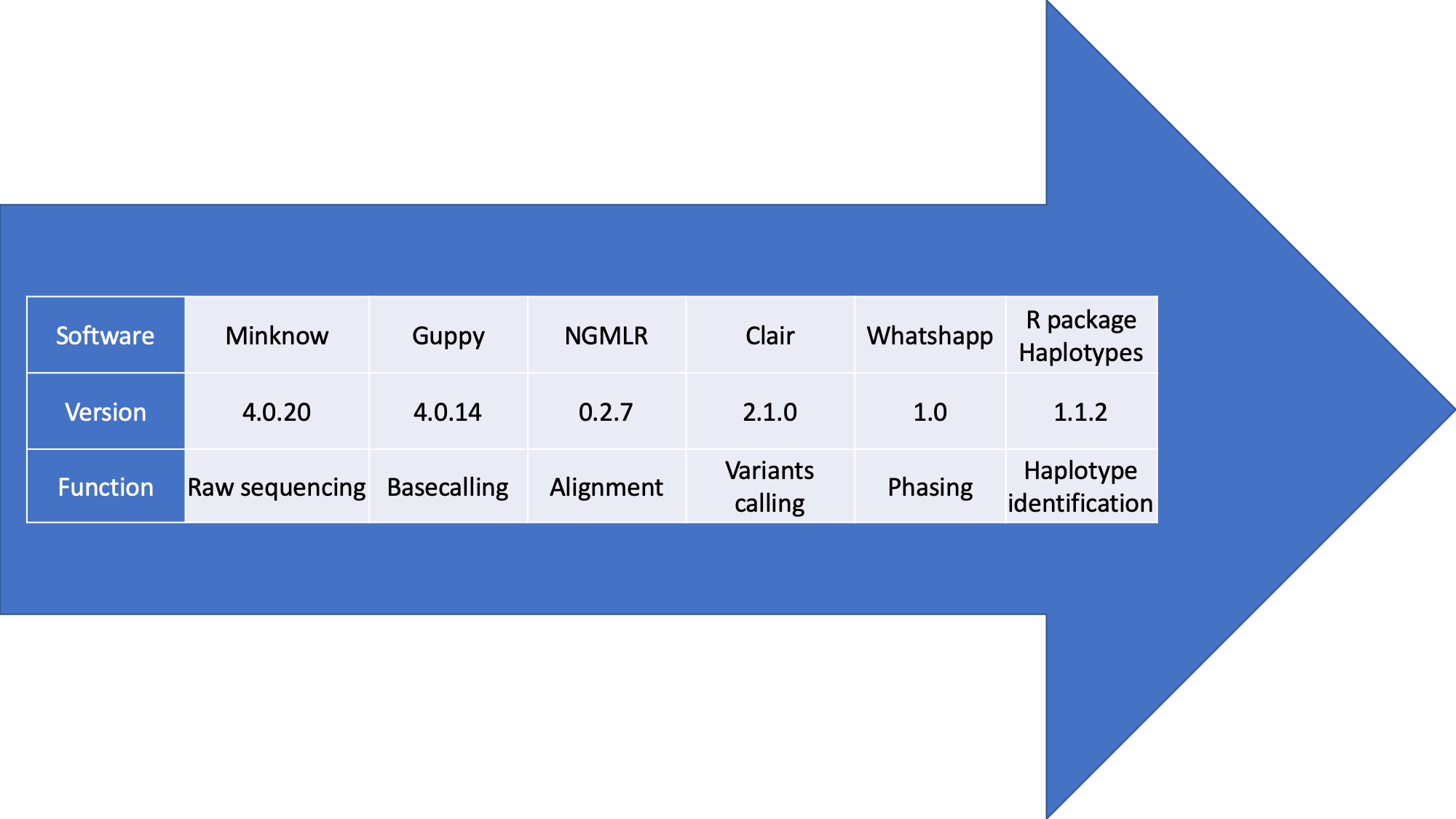


**Supplementary Figure 2:** Visual representation of the individual haplotypes identified in the RAPSODI cohort

**
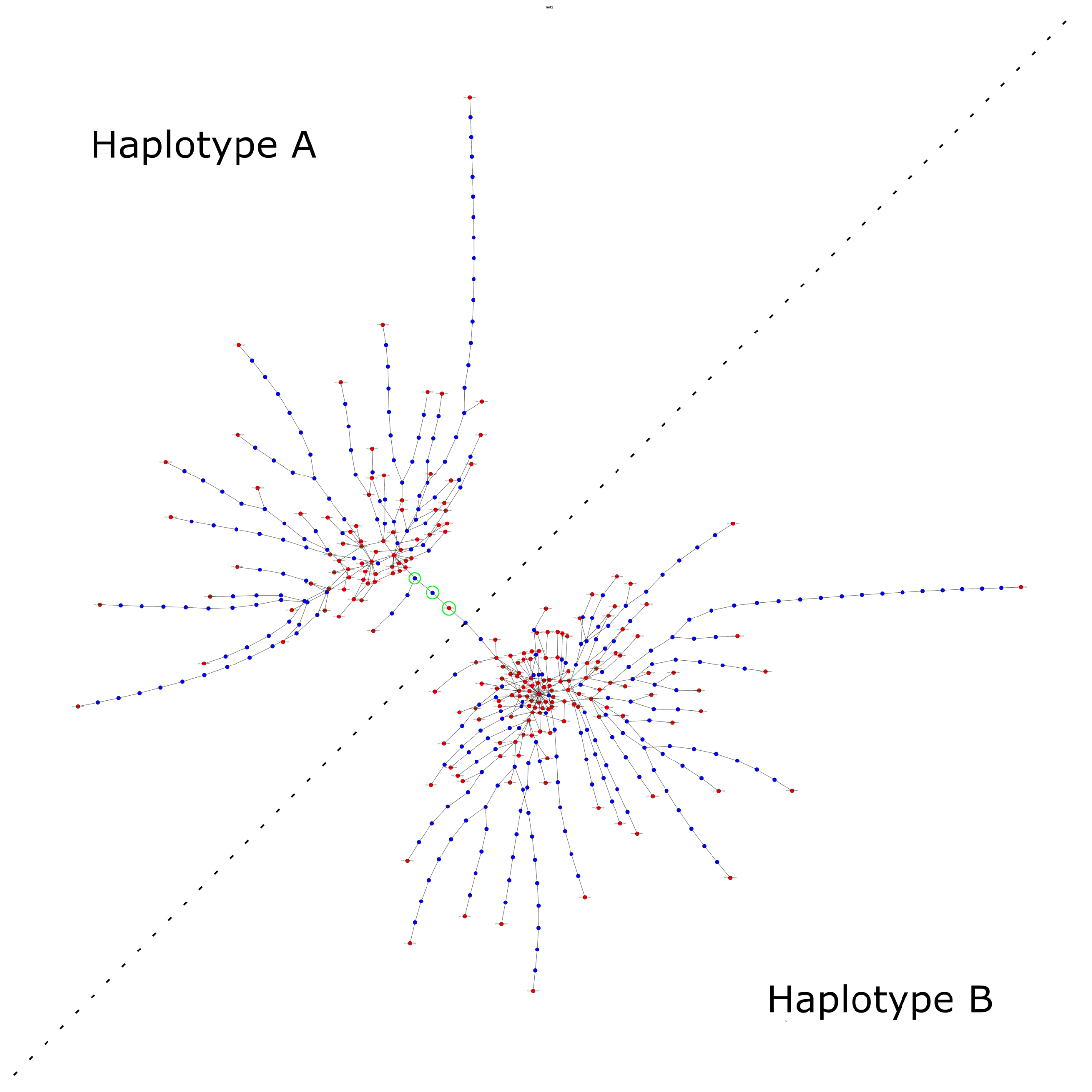
**

Each dot represents 1 single nucleotide polymorphic site. The red dots represent observed unique haplotypes, the blue dots hypothetical haplotypes. The green circles highlight the position where the 3 SNPs defining the 2 main haplotypes appear.

**Supplementary table 1:** Ethnic background in the RAPSODI cohort

|  | Homozygous haplotype A | Heterozygous | Homozygous haplotype B | Other | Total |
| --- | --- | --- | --- | --- | --- |
| White UK | 6 | 28 | 45 | 4 | 83 |
| Ashkenazi Jewish | 0 | 1 | 2 | 0 | 3 |
| White and Asian | 0 | 1 | 0 | 0 | 1 |
| Any other White Background | 4 | 3 | 5 | 1 | 13 |

**Supplementary table 2:** Ethnic background in the AMP-PD cohort

|  | Homozygous haplotype A | Heterozygous | Homozygous haplotype B | Other | Total |
| --- | --- | --- | --- | --- | --- |
| White | 115 | 504 | 615 | 5 | 1239 |
| American or Alaskan native | 1 | 0 | 1 | 0 | 2 |
| Asian | 3 | 7 | 3 | 0 | 13 |
| Black or African American | 5 | 6 | 4 | 0 | 15 |
| Multiracial | 5 | 11 | 12 | 0 | 28 |
| Other | 1 | 7 | 3 | 0 | 11 |
| Unknown | 1 | 5 | 3 | 0 | 9 |
